# VarWatch—A stand-alone software tool for variant matching

**DOI:** 10.1371/journal.pone.0215618

**Published:** 2019-04-25

**Authors:** Broder Fredrich, Marcus Schmöhl, Olaf Junge, Sven Gundlach, David Ellinghaus, Arne Pfeufer, Thomas Bettecken, Roman Siddiqui, Andre Franke, Thomas F. Wienker, Marc P. Hoeppner, Michael Krawczak

**Affiliations:** 1 Institute of Clinical Molecular Biology, Kiel University, University Hospital Schleswig-Holstein, Kiel, Germany; 2 Institute of Medical Informatics and Statistics, Kiel University, University Hospital Schleswig-Holstein, Kiel, Germany; 3 Humangenetische Praxis PD Dr. Pfeufer, München, Germany; 4 MVZ für Molekulardiagnostik GmbH, München, Germany; 5 Myriad GmbH, Martinsried, Germany; 6 TMF – Technologie- und Methodenplattform für die vernetzte medizinische Forschung e.V., Berlin, Germany; 7 Max Planck Institute for Molecular Genetics, Berlin, Germany; University of Helsinki, FINLAND

## Abstract

Massively parallel DNA sequencing of clinical samples holds great promise for the gene-based diagnosis of human inherited diseases because it allows rapid detection of putatively causative mutations at genome-wide level. Without additional evidence complementing their initial bioinformatics evaluation, however, the clinical relevance of such candidate genetic variants often remains unclear. In consequence, dedicated ‘matching’ services have been established in recent years that aim at the discovery of other, comparable case reports to facilitate individual diagnoses. However, legal concerns have been raised about the global sharing of genetic data, particularly in Europe where the recently enacted General Data Protection Regulation EU-2016/679 classifies genetic data as highly sensitive. Hence, unrestricted sharing of genetic data from clinical cases on platforms outside the national jurisdiction increasingly may be perceived as problematic. To allow collaborative data producers, particularly large consortia of diagnostic laboratories, to acknowledge these concerns while still practicing efficient case matching internally, novel tools are required. To this end, we developed VarWatch, an easy-to-deploy and highly scalable case matching software that provides users with comprehensive programmatic tools and a user-friendly interface to fulfil said purpose.

## Introduction

In recent years, high-throughput DNA sequencing of clinical samples has become routine practice in the diagnosis of human inherited diseases with monogenic etiology. In consequence, a rapidly growing number of genetic variants are being discovered for which a causative role in a given disease phenotype may be suspected. The actual establishment of causality is however difficult, particularly for rare diseases, and depends upon either functional experiments or smart and comprehensive data sharing. The former is usually highly demanding and, hence, prohibitive unless the disease in question is clearly defined, sufficiently frequent and has a reliable functional test available. An example of this type of condition is provided by hereditary breast and ovarian cancer (HBOC), where exhaustive artificial mutants of the BRCA1 gene were recently generated by saturated genome editing and successfully tested for their functional effects [1]. Although this method holds great promise, diseases like HBOC are still an exception, rather than the rule, in terms of feasibility. The second approach to infer causality (i.e. the sharing of data) aims at identifying one or more independent cases with an identical or a closely related combination of variant and phenotype. Historically, such exchange of information has been facilitated by word-of-mouth, scientific journals and public databases. Owing to the rapid growth of information generated, however, these traditional routes of communication are becoming more and more inadequate for timely data dissemination and access, particularly with a view to the necessary follow-up of unsolved cases.

In view of the above, there is growing need in medical genetics for the automated, un-supervised detection of significant similarities between cases—a process commonly referred to as ‘matching’. Responding to this need, several platforms have been established in the past that allow users to compare own variant (and phenotype) information to other reports submitted to the same service before. These platforms are usually web-based and, although hosted by strong stakeholder groups mostly from the US and Canada [2,3], offer access to registered users worldwide. More recently, some of these matching platforms have joined forces in an international network called ‘Matchmaker Exchange’ [4].

Notwithstanding its great potential, however, wide sharing of genetic data may not necessarily stand at the beginning of all instances of clinical DNA sequencing. With local databases likely to grow at rates inversely related to the decrease in sequencing costs, prior review of potential matches in own or collaborative patient cohorts that passed standardized genotyping and phenotyping workflows may instead be seen as prudent before sharing candidate variants with others. Such staged approaches could be meaningful for efficiently guiding further diagnostic activities or when used as means of internal quality control.

Moreover, the recently enacted EU General Data Protection Regulation EU-2016/679 (GDPR) not only raised public awareness of data privacy issues considerably but also heavily penalizes infringements of the stipulations made. Genetic data in particular have been defined as highly sensitive, thereby subjecting its potential use and dissemination to manifold restrictions. Given the sometimes vague wording of the GDPR and the lack of clear guidance as to what should be considered best practice, however, some uncertainty regarding the permitted level of data sharing prevails. Moreover, as more and more bioinformatics services are moving into the cloud, it is becoming increasingly difficult to decide the relevant legal framework. In the context of matching platforms, this problem is aggravated further by the potential interconnectivity of the services which allows even formally anonymized patient information to enter other jurisdictions and, usually inadvertently, the public domain.

One way to address both of the above challenges would be by hosting own matching platforms that implement all functionalities of the existing services at local level, but putting use and access under the control of the users. Given the fact that such independent solutions only make sense if sufficiently large volumes of data are being integrated, they are best suited for consortia of data producers. Here we present VarWatch, a software tool that was specifically developed to facilitate variant- and phenotype-based case matching between closely collaborating medical institutions in a dedicated network.

## Main text

### Design overview

**Fig 1:**
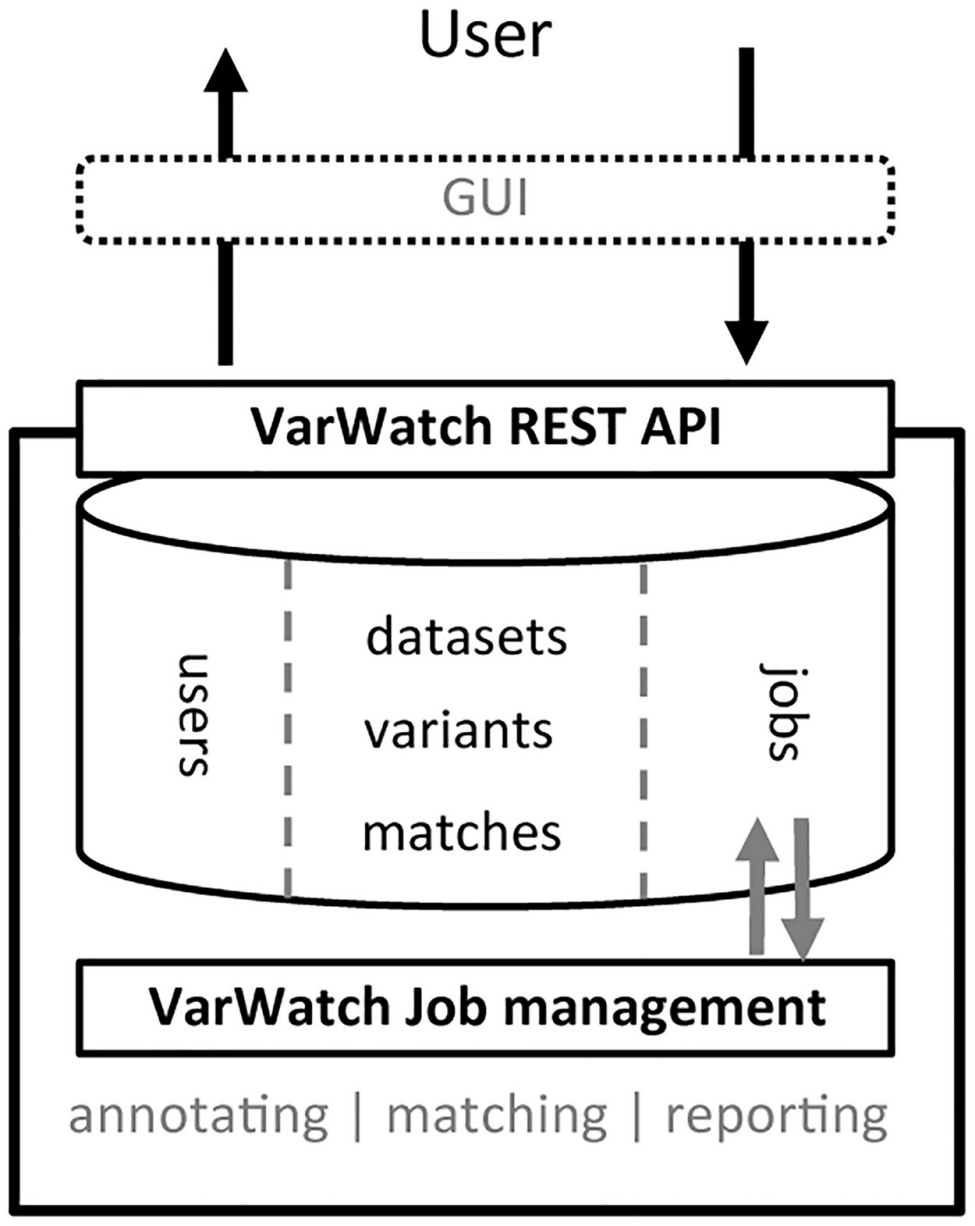
Design of VarWatch. VarWatch consists of three main building blocks, namely (i) a relational database that contains information on registered users, (ii) the watch list comprising the submitted case reports and (iii) a job management component to internally schedule the necessary processing steps, including variant annotation and matching. Users can interact with VarWatch either directly through the available REST (representational state transfer, https://restfulapi.net/) programming interface, or via a graphical web-hosted user interface.

VarWatch comprises several building blocks required for the modular implementation of a matching service (Fig 1). At its very core, VarWatch is a database of case descriptions that combine a set of candidate genetic variants with patient-specific phenotype data. Unlike traditional databases, however, VarWatch is a ‘variant watch list’ and is not meant to be queried directly. Instead, newly submitted variants are automatically annotated in terms of both their genomic context and potential deleterious effects, followed by the comparison of these descriptors and the associated phenotype(s) to all other entries in the watch list. Any ‘match’ deemed relevant by the system is reported back to the original submitter(s) of the matching entries and to the owner of the new case. This give-and-take design ensures that (i) no data are unnecessarily exposed and (ii) the watch list grows with every single use, thereby increasing the chance for future matches.

### Data definition

VarWatch defines a case by a set of ‘in limbo’ genetic variants from panel, exome or whole genome DNA sequencing that were identified in a given patient, and that have a suspected, yet unconfirmed, role in the respective disease. This implies that (i) the data have undergone some form of pre-processing or filtering by the submitter and (ii) do not include variants already present in publicly available databases. Adherence to this policy avoids over-populating of the watch list with presumably irrelevant data, which could eventually lead to non-specific (i.e. false positive) matches.

VarWatch stores variant locations in the form of genomic coordinates according to current reference assembly GRCh38. This way, the system adopts a standard that has been set by other genomic databases, including EnsEMBL [5] and UCSC [6], and by the data models of the Global Alliance for Global Health (GA4GH). VarWatch supports common input formats for variant description, specifically VCF (https://github.com/samtools/hts-specs) and HGVS [7], but also accepts a generic format specifically designed for manual input through the VarWatch web interface. If genomic coordinates are being used, the underlying reference assembly version needs to be specified (GRCh37 or GRCh38). The only additional information required for submission is at least one phenotype description according to the Human Phenotype Ontology (HPO) [8]. Optionally, VarWatch also accepts information on the suspected mode of inheritance and the age of onset of the patient. All other necessary information, including the identity of the affected gene(s) and the predicted pathogenic effect, is derived internally to ensure consistency (see below). Patient-related information not listed here remains with the diagnostician in order to minimize data privacy concerns.

### Access and security

**Fig 2:**
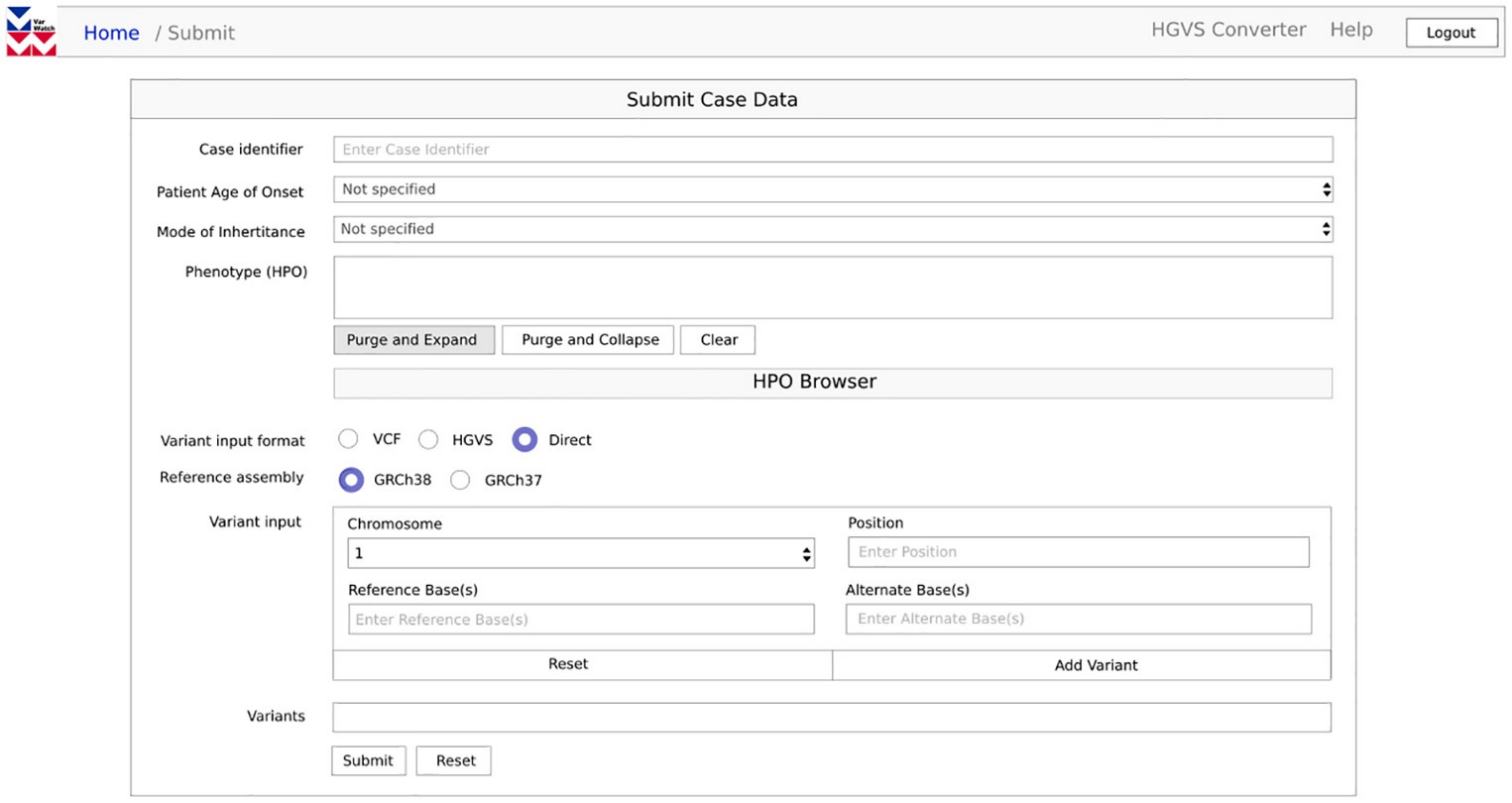
VarWatch user interface. VarWatch offers an intuitive user interface that can be hosted locally through a web server. Available functions include the upload of case reports, additional data annotation using a newly developed phenotype browser, monitoring of active submissions and the access to user data.

Access to VarWatch is provided to registered users in two ways: For programmatic access, e.g. as part of a private data processing infrastructure, a REST application programming interface (API) can be used that provides all relevant functions of the service, including authentication, data submission and matching status of individual variants as well as the retraction of submissions. Each transaction involves a user- and session-specific access token for secure data transmission, based upon an authentication system employing the OAuth2 standard (https://oauth.net/2/). Alternatively, a web-hosted graphical user interface (GUI, Fig 2) developed on top of the REST API can be used to submit new cases manually, to monitor active queries, and to access and update personal information, including a customized match notification policy.

Data protection issues are addressed at several levels in VarWatch. First, the service can be hosted on a secured server behind a firewall and/or within a specified network or IP range to prevent unauthorized access. Second, communication with VarWatch is secured by the use of transaction tokens (see above) that can be combined with a valid SSL certificate for encrypted data transfer. Finally, programmatic access to VarWatch using a scripting language requires use of a ‘client’ token, in addition to the transaction tokens. Client tokesn must be requested manually from the server operator. Without a client token, any communication attempt is rejected irrespective of whether valid user credentials were supplied.

### Data submission

New cases are submitted to VarWatch either through the API or via the web interface. Programmatically, VarWatch uses JSON-type input, drawing upon the data exchange format of GA4GH (http://ga4gh-schemas.readthedocs.io/en/latest/). Variants may be included in a submission either as individual JSON objects, a VCF-formatted file or a list of HGVS strings. Users must also provide information about the associated phenotype, encoded using the HPO, and specify the genome assembly (GRCh37/hg19 or GRCh38/hg38) upon which the variant information is based.

### Data processing

Internal processing in VarWatch draws upon a scalable architecture in which each step can be assigned prerequisites and dependencies, similar to tools like GNU Make (https://www.gnu.org/software/make/). This design allows VarWatch to be run in highly parallelized fashion, with individual pieces of information updated at runtime. VarWatch uses a relational database to define jobs that are processed by autonomous workers. Workers have a finite life span and are created by a ‘master’, based upon the current load of the system. A worker can accept jobs from a ‘blackboard’, initiate them (on a single machine or a distributed compute cluster) and report their progress to the blackboard. Each worker can only accept one job at a time. Once a job has been finished and the information written to the blackboard, one or more dependent job(s) may be scheduled. Job distribution and load balancing is implemented using the popular slurm scheduler (https://slurm.schedmd.com/) which means that, theoretically, VarWatch can be scaled out on a distributed compute system.

**Fig 3:**
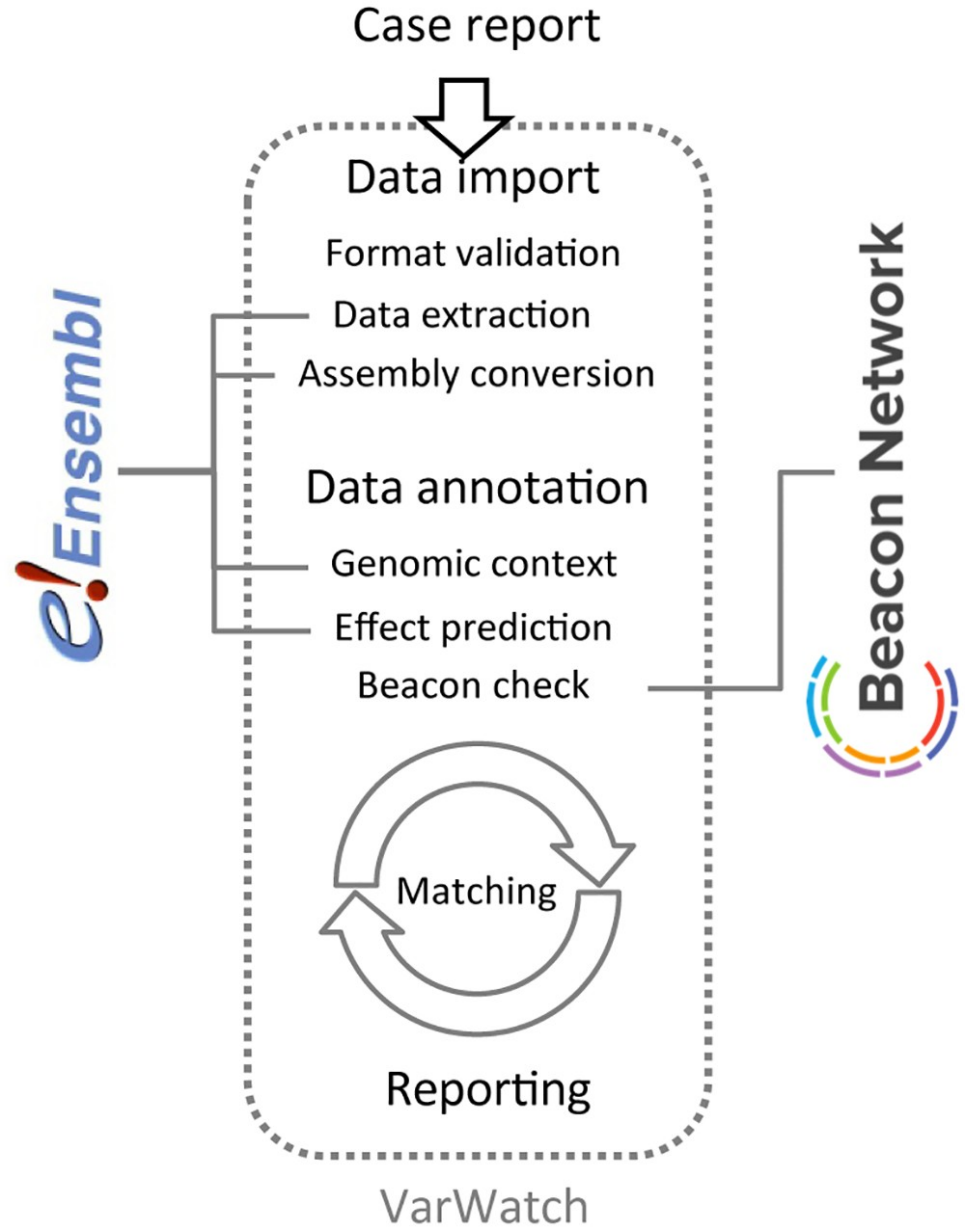
Internal VarWatch workflow. VarWatch processes newly submitted cases in several steps. First, the data are validated, extracted and left-normalized into the internal format, followed by conversion so as to match the current human reference assembly, if necessary. Next, individual variants are annotated by their genomic context and their effect(s) on overlapping transcripts. These steps are performed using the EnsEMBL REST API. Finally, variants are checked against external reference databases (using the GA4GH beacon network) to establish whether they have been publicized before.

A newly submitted case runs through several distinct processing steps (Fig 3). First, a basic sanity check is performed to verify that the data format is VarWatch-compliant and does not lack mandatory components. Next, the variants are extracted into the internal data structure of VarWatch, including conversion of HGVS strings into a proper genomic, VCF-like representation (using the EnsEMBL REST API) and transformation of variant coordinates into the current GRCh38 reference, if necessary. Left normalization [9] ensures that all variants are stored in their minimal representation. Should any of these steps fail, the respective variants are removed from the dataset and added to a processing trace for manual follow-up by the submitter.

The final set of variants is checked for their presence in external databases using the GA4GH beacon API [10] which provides simple yes/no answers to this type of query. Any hit will be logged to inform the submitter about the fact that the respective variant has already been publicized. Finally, each variant is analyzed and annotated further using the variant effect predictor (EnsEMBL REST API) to assess its genomic context and to determine its potential deleterious effects on overlapping transcripts. Optionally, the annotations provided by the VEP may be used for additional filtering such as, for example, curtailing the minor allele frequency or the predicted impact on transcript function (HIGH, MODERATE, etc.), or by defining whether transcripts must be protein-coding or canonical (i.e. are recognized as the major isoform of a given locus). Variants lacking a predicted deleterious effect or failing any of the filters are excluded from subsequent matching.

### Matching

**Fig 4:**
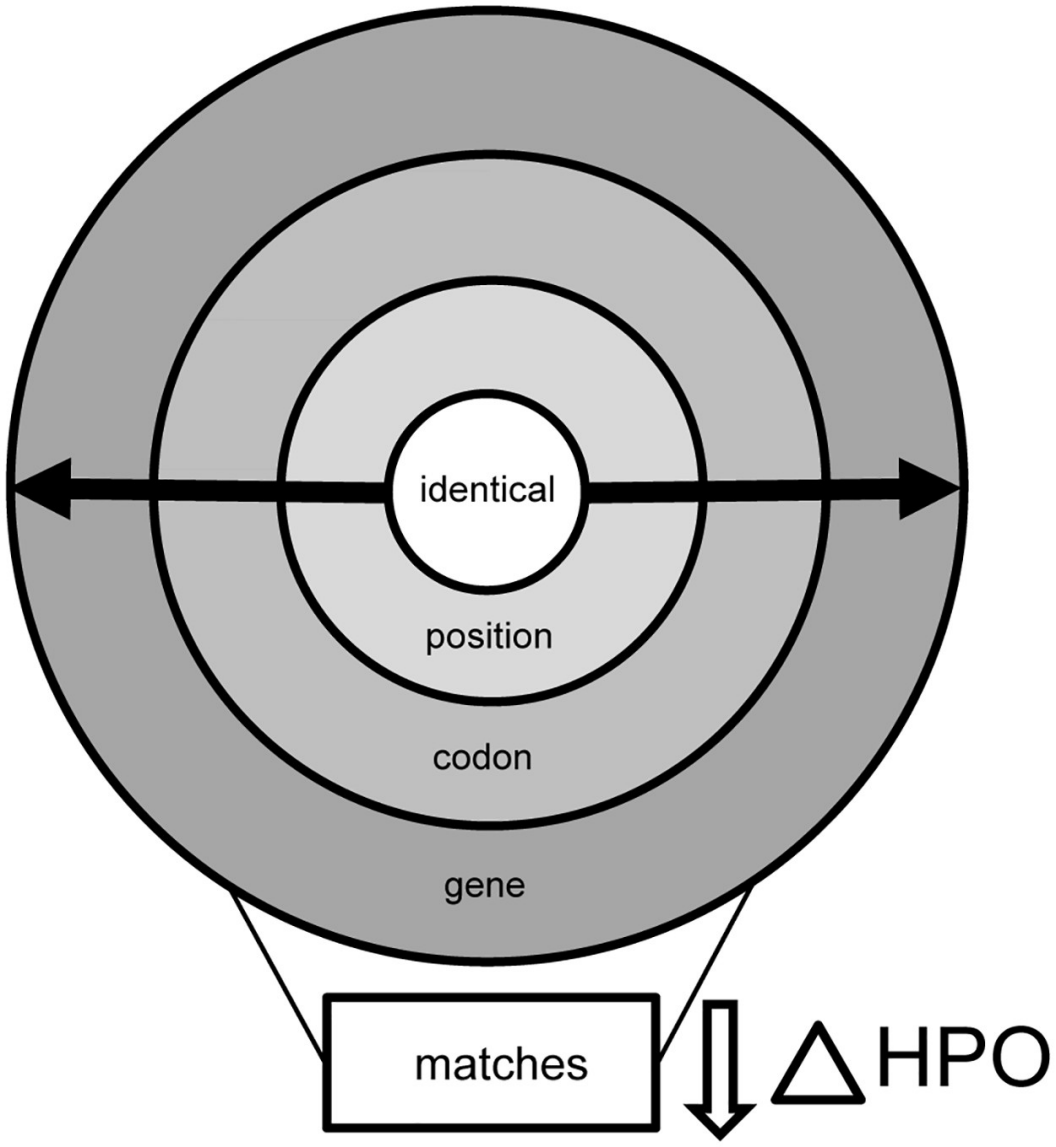
VarWatch matching. VarWatch follows a multi-level matching approach where identical variants score highest, followed by variants in the same position, then the same codon and finally the same gene. Data in each matching category are then sorted by their HPO distance to the case of interest. Finally, the matches are pruned to yield a final set of five ‘top hits’ (number configurable).

The core functionality of VarWatch is the continuous screening of the variant watch list for similarities of potential diagnostic value. To this end, the system uses a two-dimensional matching algorithm that takes both the genomic context and the phenotypic descriptor(s) of the variants in question into account (Fig 4). In a given case, pairs of newly submitted and old variant are first ranked according to their genomic proximity. Pairs of identical variants score highest, followed by pairs of different variants at the same genomic position, then same codon and finally same gene. Within each of these four proximity categories, variants are next sorted according to their phenotypic distance as quantified by a measure that exploits the ontological structure of HPO.

Several algorithms have been proposed in the past to quantify similarities in ontologies, including node-based methods like the Resnik [11] or Lin [12] distance, and different edge-based approaches [13,14]. In the case of the HPO, the definition of inter-node distances would have to draw upon the information content of each HPO term as calculated from the distribution of HPO-based phenotype descriptions in a suitable population database. However, this distribution would obviously depend upon the quality of the phenotype annotation in, and the nature of, the respective population. Consequently, the distances derived by this approach may change when the underlying reference data change.

In contrast, edge-based methods usually quantify similarities based upon the topology of a network alone, counting the number of nodes separating two terms. Although this approach avoids the population-dependency of node-based methods, it disregards annotation accuracy and is therefore potentially less specific. For example, if a submission contains the term ‘abnormal joint morphology’ and VarWatch finds two genetically equivalent matches annotated as ‘abnormal appendicular skeleton morphology’ and ‘abnormality of the lower limb’, respectively, then the first match will outrank the second because the corresponding phenotype is located at a shallower node of the ontology.

To compensate this potential drawback, VarWatch has implemented an edge-based concept of similarity that rewards precise (i.e. deep-rooted) annotations and, at the same time, is fast enough to allow rapid work-up of many queries even to a large watch list. When P(c,r) denotes the set of all different paths from HPO term c to ontology root r, then phenotypic similarity is defined as

$$\forall p\in P(c,r),p^{\prime }\in P^{\prime }(c^{\prime },r)\;sim(c,c^{\prime })=\begin{array}{c} Max\\ p\in P,p^{\prime }\in P^{\prime } \end{array}\left\{\frac{\left|p\cap p^{\prime }\right|}{\left|p\cup p^{\prime }\right|}\right\}$$

Usually, each VarWatch submission or entry is annotated with at least one HPO term. The overall similarity between any two entries, d and d’, is thus defined as

$$sim(d,d\prime )=\frac{1}{2*|d|}*\left[\sum_{c\in d}\begin{array}{c} Max\\ c\prime \in d\prime \end{array}\left\{sim(c,c\prime )\right\}\right]+\frac{1}{2*|d\prime |}*\left[\sum_{c\prime \in d\prime }\begin{array}{c} Max\\ c\in d \end{array}\left\{sim(c,c^{\prime })\right\}\right]$$

Each time the HPO is updated, all possible paths from an HPO term to the root (n = 89,961 at the time of writing) are pre-calculated and stored. For actual HPO similarity calculation in VarWatch, all paths are loaded into the main memory (where they require a few megabytes only). As a result, this set operation-based approach reduces the computational load per variant pair to a degree that allows the service to be scaled up to very large datasets.

### Availability and deployment

VarWatch is implemented in Java and is accessible at https://github.com/broderF/varwatch. For easy deployment to a wide range of systems, the service is available as a Docker container comprising the internal database, programmatic interface and web-based GUI. Since VarWatch extracts information from other web-resources during data processing, a stable internet connection is required for operation. Specific characteristics of the service such as, for example, the matching rule and the set of external databases queried, can be customized through a configuration file. A full documentation of the deployment process and the set-up options is available on github.

### Ethical compliance

VarWatch is a tool for local deployment and use that relies on highly reduced genetic data and phenotypes. Although the tool aims at minimizing data protection concerns ‘by design’, it is the responsibility of the user to ensure that processing genetic and phenotypic information in VarWatch is compliant with relevant data protection legislation.

## Discussion

With the advent of cost- and time-efficient means of primary data generation, particularly next generation DNA sequencing, turning the available genetic information into predictors of human disease has become a key concern of medical genetics research and care. The actual challenges, however, differ notably between the different types of genetic etiology involved: Whilst common complex phenotypes require transformation of the weak to modest statistical genotype associations observed in large studies into an understanding of biological mechanisms, the small data basis usually characterizing monogenic diseases calls for better means of collaboration and communication. Since many rare genetic diseases follow a simple mode of inheritance, there is reason to hope that an accumulation of independent albeit clinically comparable cases facilitates rapid pinpointing of the underlying genetic mechanisms. Against this background, the emergence of dedicated international matching platforms such as Phenomecentral [2] as Genematcher [3] has been an invaluable step forward towards the swift and comprehensive, ideally world-wide, connection of medical genetic case reports.

The existing matching platforms try to address data protection concerns by reducing the amount of externalized information to the minimum required. This notwithstanding, the scope and policy of data sharing adopted by these platforms may leave potential users in doubt as to whether their own interests and those of their patients are sufficiently safeguarded. Therefore, we are convinced that many diagnostic companies or research consortia would also contemplate interposition of a local platform, either as a safe component of their primary data processing pipeline or to enable exclusive, exploratory research of own resources. Moreover, operation of a focused matching service ‘under one’s own management’ allows the participating institutions to better tailor the handling of data privacy protection and intellectual property right issues to their own needs, not least in relation to other matching platforms. In summary, adding an intermediate layer of data handling and exploitation like VarWatch is meant to complement and support, not to question or to undermine, the sharing of well-founded candidate variants at higher levels.

The VarWatch software is open-source, flexible and modular, thereby providing users with a transparent, versatile and easy-to-use platform for variant matching. At the same time, VarWatch is highly scalable and can cope with large datasets provided that it is implemented on appropriate computing infrastructure. Hence, large laboratory consortia in particular should be able to benefit from integrating VarWatch into their data processing and analysis workflows. Moreover, since all VarWatch source code is freely available, interested users have ample opportunity to adapt the system to their own technical and organizational requirements, including the communication with external partners such as other matching platforms or data repositories.

At first glance, the development of stand-alone software like VarWatch may seem unreasonable and unnecessary because it apparently contradicts the idea of data sharing as a basis of successful variant matching. However, as was pointed out above, operation of a local platform does not necessarily imply that such a platform must exclude exchange of information with third parties. On the contrary, by collating sensitive data from different sources at an intermediate level, VarWatch not only provides matching functionality internally, depending upon the size of the connected resources, but also facilitates the coordinated implementation of an exchange with others under the network’s own terms and conditions. Seen from this side, the use of VarWatch may create a win-win situation for both, local users and a broader community.
